# Editorial: Rising stars in visual neuroscience: 2022

**DOI:** 10.3389/fnins.2024.1418533

**Published:** 2024-05-02

**Authors:** Kimberly Meier, Dorita H. F. Chang, Alexandre Reynaud, Krista R. Kelly

**Affiliations:** ^1^College of Optometry, University of Houston, Houston, TX, United States; ^2^Department of Psychology, The University of Hong Kong, Pok Fu Lam, Hong Kong SAR, China; ^3^Research Institute of the McGill University Health Center, McGill University, Montréal, QC, Canada; ^4^School of Optometry and Vision Science, University of Waterloo, Waterloo, ON, Canada

**Keywords:** vision, neurosciences, visual neuroscience, multisensory, translational

We are delighted to present the Rising Stars in Visual Neuroscience 2022 article Research Topic. This Research Topic showcases the high-quality work of emerging leaders across all domains of Visual Neuroscience research. The work featured in this Research Topic spans the entire breadth of visual neuroscience, in both theme and methodology, from the neurophysiology of the retina to behavior and computational modeling, and from fundamental research to clinical translation. The works present advances in theory, experiment, and methodology with applications to compelling problems.

Xu and Beyeler present a biophysically detailed *in silico* model of retinal degeneration that simulates the network-level response to both light and electrical stimulation as a function of disease progression. Notably, their model is the first to incorporate the global retinal remodeling effects known to occur in these degenerative diseases. The model reproduces common characteristics of retinal ganglion cell activity in the degenerated retina, such as hyperactivity and increased electrical thresholds, but also reveals nuanced differences in the effects of disease and stimulation on ON and OFF cells, generating testable predictions about underlying neuroanatomical mechanisms. Their findings further our understanding of visual processing in the retina and may inform the design and application of retinal prostheses.

Wang et al. studied the impact of sensory uncertainty and feedback on decision strategies and performance in a sequence prediction task. By training participants on sequences of symbols determined by first-order Markov models and asking them to indicate which symbol they expected to follow each sequence while manipulating its uncertainty, they were able to track decision strategies as they developed over time. Their results indicate that executive cognitive functions such as selective attention may account for the individual variability in strategy and structure learning ability. Individuals adapt their decision strategy closer to probability maximization, reducing uncertainty in temporal sequences and improving their ability to learn predictive statistics in variable environments. Overall, this work underscores the adaptability of learning strategies in uncertain environments, with implications for educational approaches, cognitive training, and even clinical interventions targeting cognitive impairments.

This issue also highlights clinical/translational applications in visual neurosciences. Three studies present new research in intermodal/crossmodal integration between vision and other sensory motor systems in clinical conditions: vision, echolocation, and haptics (Teng et al.), vision and audition (Moro et al.), and vision and motor control (Kartha et al.).

Active echolocation allows blind individuals to explore their surroundings via self-generated sounds such as finger snaps or mouth clicks, similarly to dolphins and other echo-locating animals. Teng et al. investigated the resolution of crossmodal transfer of object-level information between acoustic echoes and other senses in blind expert echo-locators and sighted novice controls. Using a match-to-sample paradigm, they showed that coarse object information transfers from auditory to haptic modalities and may be facilitated by prior object familiarity and/or material differences. They further demonstrated that difficulty in haptic discrimination may be limited by the coarse sampling resolution of echolocation itself, rather than limits in echo-haptic crossmodal transfer. Estimating the quality of echo-acoustic information that transfers to other sensory modalities may help inform the design of assistive technology for blind individuals.

Abnormal visual input during the postnatal period and prior to visual maturation is well known to bring about deficits in visual processing but may also affect the development of complementary senses. The loss of one eye early in life has previously been associated with changes in auditory and audiovisual plasticity, but little is known about such compensatory mechanisms when one eye is lost later in life, after visual system maturation. Using a paradigm based on the McGurk effect, a classic crossmodal illusion in which visual information can influence auditory perception, Moro et al. showed that people who had one eye removed late in life perceived the McGurk effect similar to binocular-viewing controls, contrary to those who had one eye removed early in life, who showed less susceptibility to this effect. This suggests that cross-modal accommodations following the loss of binocularity are dependent on the period (i.e., before or after development) during which the eye was removed. These findings set the stage for exciting investigations geared at systematically understanding the effects of vision-loss on adaptive sensorimotor compensation during key stages of development.

Finally, the last study presents a new digital tool for clinical/translational research in ultra low vision. Ultra low vision refers to profound visual impairment, but existing assessment tools for this condition provide limited information about patients' ability to perform the activities of daily living. Kartha et al. developed a new performance test in virtual reality that can be used to assess hand-eye coordination in individuals with ultra low vision, incorporating functional movements including reaching, grasping, pointing, distance and depth judgements, and reaction time. This test provides a validated outcome measure under real-life scenarios that can be used to track progression and improvement in vision restoration trials, and to monitor rehabilitation outcomes in people with ultra low vision.

This Research Topic presents a very wide range of findings and innovations by promising early career researchers in all fields of visual neurosciences. While future innovations in Visual Neuroscience are yet to be discovered, this Research Topic will give us a hint at whom to follow for the latest trends and advancements in the field.

## Author contributions

KM: Writing – review & editing. DC: Writing – review & editing. AR: Writing – original draft. KK: Writing – review & editing.

